# Developing trustworthy AI for climate services at scale

**DOI:** 10.1038/s44168-026-00420-z

**Published:** 2026-08-22

**Authors:** Hywel T. P. Williams, Anrijs Abele, Arjun Biswas, Fai Fung, Jason A. Lowe, Neha Mittal, Hailun X. Xie

**Affiliations:** 1 Centre for Environmental Intelligence, University of Exeter, Exeter, UK; 2 Met Office, Exeter, UK; 3 Centre for Climate Research, Singapore, Singapore; 4 University of Bristol, Bristol, UK; 5 Priestley Centre, University of Leeds, Leeds, UK

**Keywords:** Business and industry, Climate sciences, Environmental social sciences, Mathematics and computing, Scientific community, Social sciences

## Abstract

Generative AI can help scale climate services to meet growing demand. This approach is illustrated here with two prototype AI systems designed to enhance access to climate data, support decision-making, and improve efficiency. Key concerns are identified around responsibility, quality, and trustworthiness of AI outputs. Emerging best practices in system development are linked to core principles of salience, credibility, and legitimacy, to ensure alignment with user needs and societal values.

## Motivation: Can AI help to scale climate services?

Decades of climate change research have created a huge volume of knowledge and expertise^1^, yet its effective use to inform climate adaptation is challenged by a bottleneck of human labour. Climate services—the provision and use of climate change knowledge to support decision-making—are difficult to scale. Increasing unmet demand for climate-related skills in the labour market^2^ suggests there are too few climate specialists to provide in-depth guidance to the many people and organisations who must respond to climate change in the coming years. While climate mitigation and decarbonisation efforts continue, “committed warming” and observed emissions trajectories indicate around 2.5–3 °C of warming by 2100^3^. Adaptation is made more challenging by the need for localised, context-dependent, and impact-specific strategies^4^.

Meanwhile, new generative AI technologies present the intriguing possibility of partial automation of climate services, potentially removing barriers to access and improving decision-making. While conventional software tools (e.g. data platforms, decision-support tools) can help meet demand, generative AI might offer enhanced ease of use, greater autonomy and/or automation capacity, and the ability to connect diverse data formats across domains. Yet such technologies are unproven and might inadvertently weaken or disrupt the effective use of climate science if they are not deployed in a responsible manner (i.e. aligned with common responsible innovation principles of anticipation, reflection, engagement and action^5^). The climate services context brings domain-specific risks around AI systems that (e.g.) misinterpret climate science, confuse models or emissions scenarios, or do not track uncertainty. Here we consider how generative AI might help deliver climate services at scale, and the need for such methods to be trustworthy, equitable, and integrated with human workflows. We identify autonomy, flexibility and uncertainty as shared features of generative AI tools in the climate services context, as well as some emerging best practices to improve system quality.

## Examples of existing AI tools for accessing climate information

“Generative AI” refers to the ability of some kinds of machine learning system, most commonly large language models (LLMs, very large neural network models trained on enormous text datasets), to create multimodal content (e.g. text, images, video, audio) that is fast becoming indistinguishable from human-created content. Such models are the basis for popular AI applications like chatbots, image-generators, virtual assistants and analytical tools, which are beginning to appear in the climate services domain (e.g. refs. ^6–9^). Subject to critical evaluation, such tools might be integrated with climate services workflows to help synthesise information and support human decision-making.

**Fig. 1:**
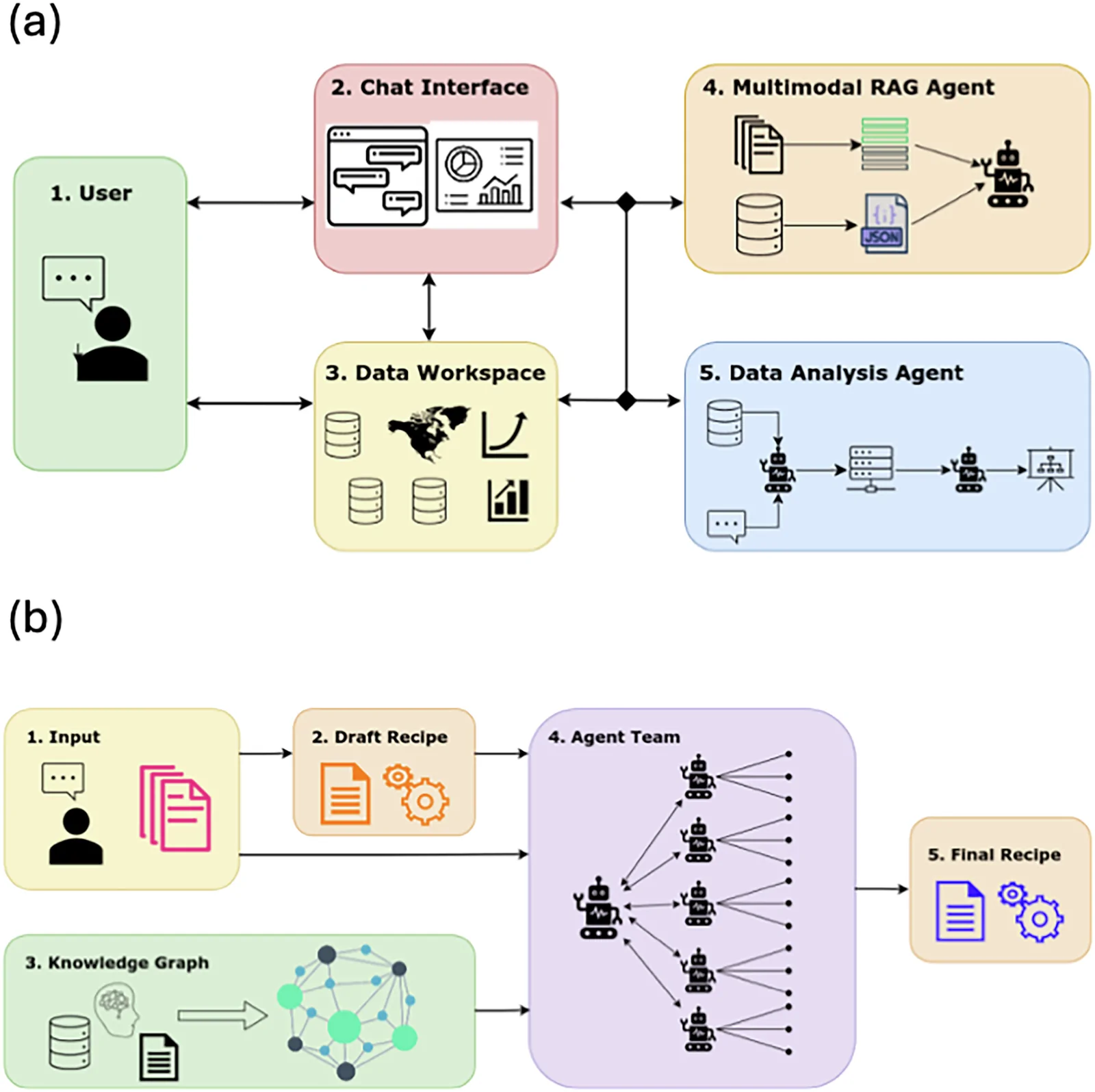
Schematic diagrams showing workflows for illustrative examples of agentic AI tools for climate services. **a** Multimodal chatbot and data analysis tool for UKCP. 1. User interacts with chat interface and data workspace. 2. Chat interface provides multimodal outputs and data visualisation. 3. Data workspace holds extracted datasets, analysis/visualisations, and chat history. 4. RAG agent supports multimodal question-answering based on UKCP archive. 5. Data agent enables data extraction and generates code to perform analysis/visualisation. **b** Agentic workflow for generative climate service recipes. 1. User provides input: templated task/query and project description. 2. Reasoning LLM is prompted to create a “naïve” draft recipe. 3. Knowledge graph collates climate service case studies and climate models/datasets. 4. Multiple LLM agents (supervisor & specialists) augment/improve naïve recipe with specific climate models/datasets/procedures, constrained by knowledge graph. 5. Output is an actionable “final” recipe provided back to the human user.

The AI tools so far reported in the climate services literature (e.g. refs. ^6–9^) have diverse purposes, datasets, and approaches to LLM integration. As an illustration from a UK perspective, here we consider two such applications from our own work. Figure 1 shows schematics for a chatbot designed to communicate information from the UK Climate Projections (UKCP18) (Fig. 1a) and for a tool for generating climate service “recipes” based on a large evidence base of climate models and adaptation solutions (Fig. 1b). Both tools adopt an “agentic” approach (i.e. modular systems which decompose the larger task into smaller sub-tasks that are each handled by semi-autonomous LLM-based agents). Both also use “human-in-the-loop” mechanisms for resolution of ambiguities. Both were co-created with end users at the UK Met Office, with users involved in shaping and evaluating the systems.

UKCP18 was produced by the UK Met Office to provide robust climate projections and aid planning and decision-making for the UK until 2100. It consists of scientific reports (containing text, images, tables and graphs), large numerical datasets of climate model outputs (in gridded/tabular form), and various ancillary content (e.g. visual summaries, documentation). Despite many applications, many users highlight difficulties in applying UKCP18 outputs without technical support^10^. Our experimental chatbot (Fig. 1a) seeks to overcome this usage barrier by using natural language interactions to provide user-friendly and fast access to UKCP18 data/information, with question-answering, data retrieval, and data analysis functionalities. It uses retrieval-augmented generation (RAG)^8^ to ground LLM responses in the UKCP18 knowledge base and reduce the risks of inaccuracy or hallucination (generating a false or unfounded answer that is not based on real information^11^). The RAG tool is multimodal and can answer with relevant images/graphs or datasets as well as text. Human-in-the-loop validation is used when the system detects an ambiguous query, especially during data selection tasks where precision on spatial location, time period and climate variables is needed. The chatbot also supports interactive analysis of raw climate model data, generating on-the-fly code that enables users to (in natural language) select different climate variables, locations and time periods for visualisation, comparison, and statistical analyses. Many users previously required input from a (human) climate consultant to access these functions.

Our second example tool (Fig. 1b) is an agentic system designed to automatically generate climate service recipes (i.e. detailed workflows for application of climate science in a given scenario or context). This tool aims to help human climate service providers to easily identify a method for using climate data in a particular client context, reducing the time and cost of delivery. The tool combines LLM reasoning with contextual knowledge from a curated knowledge graph^12^ that maps relationships between climate variables, emission scenarios, indices, hazards, sectors, and key datasets (e.g. CORDEX, CMIP5, UKCP18). The agentic workflow begins with a description of a climate scenario; human-in-the-loop confirmation is used to augment the initial description and complete a scenario template for further processing. The architecture involves a memory-enabled supervisor agent that orchestrates multiple specialised agents to plan workflows, create draft recipes, query the knowledge graph, source external (non-climate) domain knowledge, and summarise the final output. This multi-agent approach effectively generates “recipes” that specify appropriate methodologies and datasets, with clear traceability back to the knowledge graph to encourage user trust. The approach shows good potential to increase efficiency of climate service design across multiple hazards and domains, enabling human consultants to deliver climate services more rapidly.

## Emerging best practices for trustworthy AI development

From the two examples above, and many others in the literature (e.g. refs. ^6–9,11,13^), we can identify three characteristic features of generative AI systems that are particularly relevant to the climate services context. Firstly, *autonomy*; the LLM components, and therefore the wider agentic systems, are given an amount of freedom in how they respond to a query or complete a task. Second, *flexibility*; these tools can respond effectively to a wide range of inputs and generalise to novel situations. Third, *uncertainty*; the inherent complexity and stochasticity of LLMs makes derived systems somewhat opaque, and it is hard to be sure what answer they will give to a given query. These three attributes give these systems their ability to generate useful and diverse outputs, but also introduce unpredictability and risk of inaccuracy; these are undesirable features in the context of climate services, where precise use of climate science, terminology, and data/models is essential.

Much effort in system development is expended on constraining such tools to reduce uncertainty and avoid undesirable behaviours. The examples above, and others in the literature, have done this by: (1) careful *prompt engineering* to steer the behaviour of LLMs, by both users in their queries and developers in internal system prompts; (2) *controlled access to data and resources*, such as RAG systems that force answers to be generated only from a given database; (3) embedding *transparency* in system behaviour using visible reasoning steps and traceability of responses; (4) using *human-in-the-loop* confirmation to resolve ambiguities; and (5) rigorous *evaluation* of outputs using both quantitative and qualitative metrics. Implementing these practices requires careful consideration. For example, human-in-the-loop mechanisms can only scale when the human is the end user (who is always present) rather than a developer (who is no longer involved at point of delivery). Development should also adapt to the target user audience; tools for public use might require different standards to those designed for experts. Yet ultimately, the three core features of autonomy, flexibility, and uncertainty are simultaneously the strength and the potential weakness of generative AI tools. Good engineering can improve performance and consistency, but the inherent uncertainties of such technologies suggest that careful human steering (of both development and usage) will be needed to achieve trustworthy and high-quality outcomes. Users and developers both share responsibility for the accurate generation and careful interpretation of AI system outputs.

## AI for climate services should prioritise salience, credibility and legitimacy

Beyond implementation, questions remain about whether creation of automated tools for climate services is desirable or responsible. Responsible innovation principles (e.g. the AREA framework^5^) should be followed in developing any new AI technology, but the climate domain has particular features. Climate services are delivered in complex contexts currently requiring expert human judgement; uncritical adoption of AI-generated solutions and loss of human oversight might have serious consequences. Emerging standards that seek to regularise and improve quality of climate service delivery (e.g. ref. ^14^) might be disrupted or undermined by integration of AI tools. To explore these issues, we held a UK-based stakeholder workshop (Box 1) that identified additional risks around lack of accountability, inequality of access, and value alignment (Table 1). These views, while exploratory and drawn from a UK-only sample, align with previously reported concerns in the climate services literature (e.g. ref. ^15^).

**Fig. 2:**
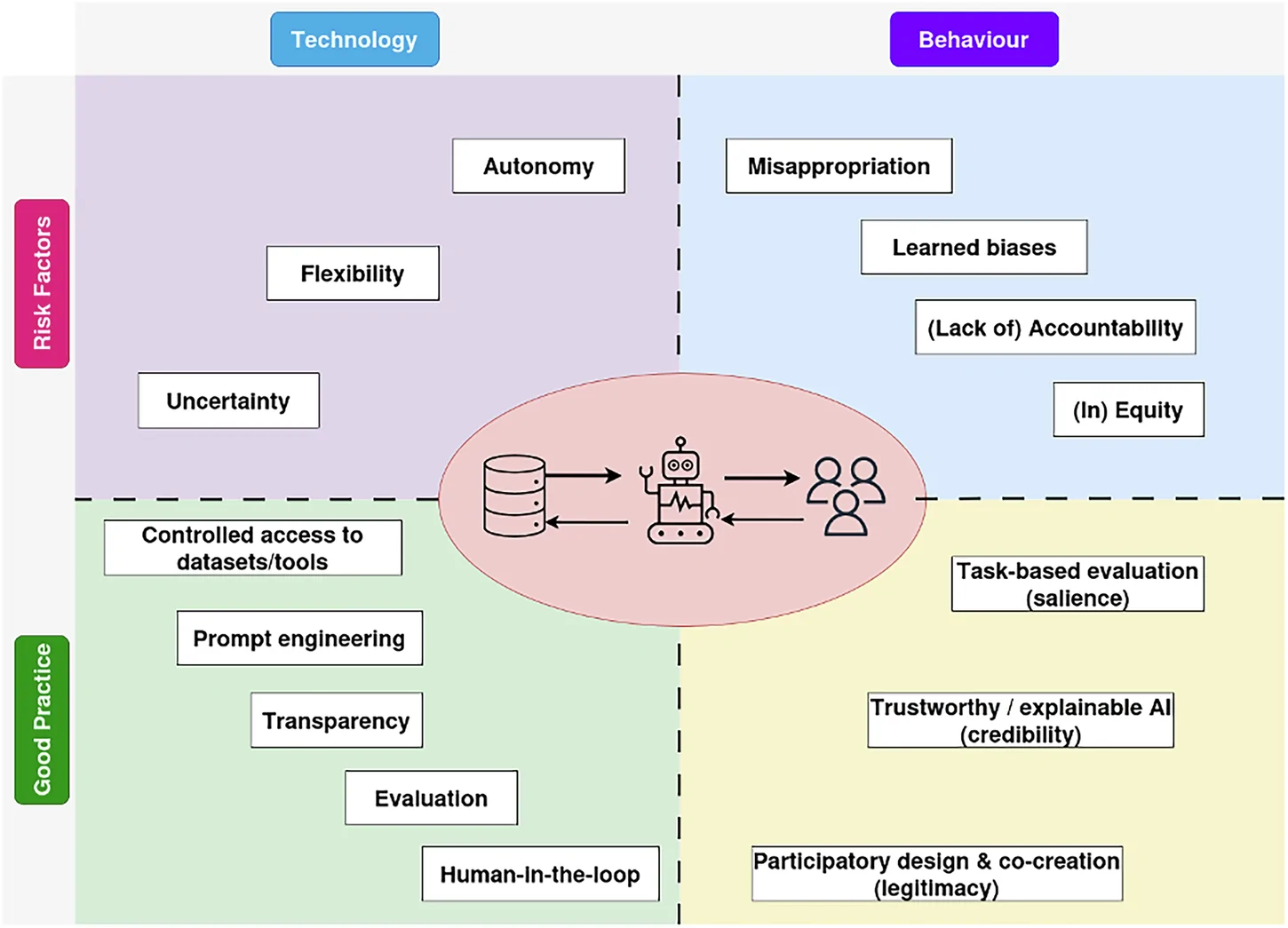
Risk factors and suggested good practices for generative AI tools for climate services.

Here we suggest some guiding principles for AI deployment in climate services (Fig. 2), framed around the foundational climate service concepts of *salience, credibility* and *legitimacy*^16^. To ensure salience, AI tools should provide climate information specific to user context and decision scenario. Furthermore, to date there has been little exploration of whether such tools are useful; future behavioural experiments under controlled conditions could evaluate whether their use improves or worsens decision-making outcomes^17^. A perspective of continuous improvement and user engagement should be adopted to ensure ongoing alignment with community/stakeholder needs.

Considering credibility, the central concerns for any new AI tool for climate services should be trust and reliability, with robust evaluation (including co-evaluation by stakeholders) to ensure outputs are accurate and consistent with established climate science. Both quantitative and qualitative evaluation approaches are valuable, while benchmarks and tests should be tailored to the specific context (ideally with engagement from stakeholders). AI tools for climate services should adopt emerging best practices for creating “trustworthy” AI systems, prioritising transparency and interpretability to avoid undesirable “black boxes” and build user confidence^18^.

To aid legitimacy, AI tools for climate services should seek to mitigate concerns about social, cultural or geographic biases, and meet common standards of political/procedural fairness. Participatory design and co-creation can help increase legitimacy by ensuring that tools reflect user needs and local contexts^16^. To aid transparency and accountability, tools should have clear governance, follow good software development practices (e.g. version control, data updating), and give clear information about ownership, construction, inputs, and underlying models, as well as responsible usage/interpretation and liability guidelines for outputs.

Box 1 **Exploring concerns around AI for climate services** A workshop held at the Met Office (Exeter, UK) in February 2024 explored AI’s impact on climate services, focusing on *legitimacy*, *credibility*, and *saliency*^16^. Thirty-four UK-based climate scientists, service providers, and AI practitioners were selected to represent the climate services community. All participants completed a pre-workshop survey to identify: (i) potential challenges and risks associated with integrating AI into climate services; and (ii) potential solutions or pathways for addressing these challenges. Then 24 participants attended in person from academia (*n* = 14), government (*n* = 4), and commercial sectors (*n* = 6). In facilitated discussions, the group considered both opportunities and risks of AI, mainly generative tools like LLMs, and proposed strategies to address potential challenges. Workshop findings were later collated from recurring themes identified across survey responses and discussion outcomes. While the UK-based group and qualitative approach limit the generality of the findings (e.g. across geographies), the workshop produced useful exploratory findings from a representative group.

Identified risks (Table 1) echoed those previously reported in climate services literature (e.g. ref. ^15^): concerns about fairness, transparency, trustworthiness, and value alignment, especially regarding which values are reflected in AI outputs. AI-specific risks included misappropriation of information, model biases, lack of accountability (of both commercial providers of LLMs and developers of AI-based climate services applications), and the potential to worsen inequalities in access to climate services (cf. the “digital divide”). Solutions such as codes of practice, accreditation, co-production, and interdisciplinarity were suggested, aligned with established best practices, showing that while generative AI introduces new issues, it also reinforces proven approaches (see Fig. 2).

**Table 1:** Selected quotes from workshop participants illustrating concerns about AI tools in climate services

*“AI becoming a ‘black box’ in which it is challenging to assess the accuracy/credibility/suitability of what comes out.”*
*“AI can find relationships that are difficult to perceive by humans but can also enhance our biases or omissions in the data that is provided.”*
*“Service providers may use reliance on AI to deny responsibility for decisions made on the basis of their services. When models that are the work of many people fail, who takes responsibility?”*
*“AI could also give more weight to experts than those with lived knowledge.”*
*“Whoever puts in the values with the system has a lot of power!”*

## Conclusion: Responsible AI can help deliver climate services

If the concerns outlined above can be addressed, generative AI technologies offer substantial potential benefits for delivering climate services at the scale needed to support the local, national and international actions needed for climate adaptation and resilience. Specialised tools could assist human practitioners to deliver projects faster, cheaper and more consistently, thereby increasing accessibility, relevance and quality for end beneficiaries. AI tools and applications could be deployed at scale via the Web, in most languages and geographic regions, with minimal cost at point of use. This would help increase access for people and organisations where language and/or resource constraints currently prevent effective uptake of climate knowledge. However, achieving these potential benefits will require careful deployment to avoid worsening digital divides (e.g. by alignment with inclusive design standards^19^) and ensure usage costs do not become prohibitive (e.g. by use of open source models). It is also important that deployment of AI for climate services does not worsen climate change; systems might be deployed on data centres using renewable energy sources and use ‘green prompting’^20^ to reduce their energy use and associated greenhouse gas emissions. Expanding our collective ability to apply climate science to real world adaptation challenges might enable people everywhere to make better decisions, as climate impacts start to affect their lives.

## Data Availability

No datasets were generated or analysed during the current study.
